# Physical Activity Surveillance in Children and Adolescents Using Smartphone Technology: Systematic Review

**DOI:** 10.2196/42461

**Published:** 2023-03-29

**Authors:** Nur Izzatun Nasriah Nasruddin, Joey Murphy, Miranda Elaine Glynis Armstrong

**Affiliations:** 1 Centre for Exercise, Nutrition and Health Sciences School for Policy Studies University of Bristol Bristol United Kingdom

**Keywords:** physical activity, surveillance, children, adolescents, smartphone technology, smartphone apps, smartphone, technology, application, database, mobile phone

## Abstract

**Background:**

Self-reported physical activity (PA) questionnaires have traditionally been used for PA surveillance in children and adolescents, especially in free-living conditions. Objective measures are more accurate at measuring PA, but high cost often creates a barrier for their use in low- and middle-income settings. The advent of smartphone technology has greatly influenced mobile health and has offered new opportunities in health research, including PA surveillance.

**Objective:**

This review aimed to systematically explore the use of smartphone technology for PA surveillance in children and adolescents, specifically focusing on the use of smartphone apps.

**Methods:**

A literature search was conducted using 5 databases (PubMed, Scopus, CINAHL, MEDLINE, and Web of Science) and Google Scholar to identify articles relevant to the topic that were published from 2008 to 2023. Articles were included if they included children and adolescents within the age range of 5 to 18 years; used smartphone technology as PA surveillance; had PA behavioral outcomes such as energy expenditure, step count, and PA levels; were written in English; and were published between 2008 and 2023.

**Results:**

We identified and analyzed 8 studies (5 cross-sectional studies and 3 cohort studies). All participants were aged 12-18 years, and all studies were conducted in high-income countries only. Participants were recruited from schools, primary care facilities, and voluntarily. Five studies used mobile apps specifically and purposely developed for the study, whereas 3 studies used mobile apps downloadable from the Apple App Store and Android Play Store. PA surveillance using these apps was conducted from 24 hours to 4 weeks.

**Conclusions:**

Evidence of PA surveillance using smartphone technology in children and adolescents was insufficient, which demonstrated the knowledge gap. Additional research is needed to further study the feasibility and validity of smartphone apps for PA surveillance among children and adolescents, especially in low- and middle-income countries.

## Introduction

### Background

Research suggests that the period of childhood and adolescence are critical in a person’s development and growth [1]. Engagement in adequate physical activity (PA) results in various health benefits for children, such as lowering the risk of childhood obesity and improving cognitive functioning [2]. Evidence has shown that children who meet PA recommendations have a healthier cardiovascular profile and are likely to become active adults as part of the behavioral carryover effects [3]. According to the World Health Organization, children and adolescents (aged 5-17 years) are recommended to do on average at least 60 minutes of moderate to vigorous PA (MVPA) per day to achieve the associated health benefits [4]. However, the latest World Health Organization report in 2019 found that approximately 80% of school-going adolescents (85% of girls and 78% of boys) aged between 11 and 17 years did not meet this recommendation [5].

Engagement in regular MVPA is associated with numerous health benefits [6,7], including positive cognitive development in children and adolescents [8,9]. Research has also shown that greater benefit may come from vigorous activity intensities, in which aerobic-based activities have the greatest health benefit in school-aged children and youth [6]. Given this, it is of paramount importance that PA in children is accurately measured and assessed to identify current PA levels, monitor compliance with PA recommendations, and assess the effectiveness of intervention programs designed to promote PA in children and adolescents [10,11]. Traditionally, PA in this age group is measured using self-report methods such as questionnaires, diaries, and activity logs as well as objective measurements such as heart rate monitoring, direct observation, doubly labeled water, pedometers, and accelerometers [10,11].

However, in recent years, there has been an increasing demand in the use of digital communication technologies in daily life [12-14]. The modernization of health services, delivery, and systems has encouraged the development of digital health, which is now considered a cornerstone in participatory or personalized health [15]. The advent of smartphones and wearable devices has greatly influenced mobile health (mHealth), offering new opportunities in health research, including PA research. These technologies offer real-time and continuous biological, behavioral, and environmental data that enable researchers to understand the etiology of health and disease as well as provide new approaches for the measurement of PA in children and adolescents [16-18]. With the evolution of technology, smartphone apps and wearable activity trackers are currently among the range of mHealth technologies used to measure PA in children and adolescents.

To date, several existing reviews have addressed the use of mHealth in PA research in adult and adolescent populations [19-26]. There are 3 reviews involving adolescents and postsecondary students to date: a study by Lee et al [21], which discussed the efficacy and efficiency of mHealth apps in PA promotion with adolescents; a study by Böhm et al [26], which focused on evaluating the effects of mHealth to increase PA outcomes among children and adolescents; and a study by Ly [23], which aimed to examine the relationships between mobile phones and PA behaviors in postsecondary or university students, with a focus on text messaging interventions. Mönninghoff et al [19] and Laranjo et al [25] aimed to understand the effects of mHealth apps such as smartphone apps and activity trackers interventions to increase PA in adults. Meanwhile, other reviews addressed the use of mHealth technology, specifically smartphone apps, to promote PA and reduce weight in adults [20] and understand the trajectory of smartphone-based interventions for PA promotion in adults and adolescents for the past 10 years [24]. Although informative and useful for addressing the promotion of PA using mHealth technologies, these reviews did not explore the use of smartphone apps for the surveillance of PA in children and adolescents.

Studies have also shown that objective measurements such as smartphone technologies have the potential to improve PA measurement [27] and potentially reduce the costs of PA surveillance when compared with traditional methods [28,29]. Face-to-face measurements are standard practice; however, in certain conditions, these methods may not be ideal. For example, the COVID-19 pandemic has affected the whole world and changed the way research has been conducted. Smartphone technology offers a different approach for PA measurements, where it allows individual users to install, configure, and run the apps of their choice [30]. These smartphone apps with built-in sensors that can monitor the duration, frequency, and intensity of PA are ideal alternatives for PA measurements and surveillance research [31]. Given this, it would be beneficial to assess whether the advancements in smartphone apps are usable and practical for PA surveillance in children and adolescents.

### Objectives

This systematic review aimed to explore the use of smartphone technology for PA surveillance among children and adolescents. This review focuses on smartphone apps for PA surveillance and whether they are built into smartphones, downloadable from app stores, or study-specific developed apps.

## Methods

This systematic review was conducted following the PRISMA (Preferred Reporting Items for Systematic Reviews and Meta-Analyses) statement [32]. The quality of each study included in this review was assessed using the Strengthening of the Reporting of Observational Studies in Epidemiology (STROBE) checklist.

### Identifying the Research Question

On the basis of the current research gap, three research questions were identified to guide this systematic review:

1. How has smartphone technology been used in the PA surveillance of children and adolescents?
2. How accurate are smartphone technology surveillance methods when compared with objective measures of PA?
3. What are potential research gaps within the existing literature requiring further research?

### Identifying Relevant Studies

A systematic literature search was performed on all articles published between January 7, 2008, and December 22, 2022. This date restriction was applied because the Apple App Store was introduced in July 2008 by Apple Inc and Google Play Store was launched into the market in 2012, after the rebranding of the Android Market [33]. Five databases were accessed (PubMed, Scopus, CINAHL, MEDLINE, and Web of Science) to find peer-reviewed publications, and Google Scholar was used to find gray literature and additional studies related to the topic. These databases were selected after consultation with a university librarian, who also provided advice on developing the search terms (Multimedia Appendix 1). The search terms used were “smartphone*” OR “smartphone app*” OR “mobile phone” OR “mobile app*” OR “smartphone technolog*” OR “mobile technolog*” AND “physical activity” OR “physical activity level” OR “step count*” OR “energy expenditure*” OR exercise AND child* OR adoles* AND measurement* OR assessment* OR surveillance. All the search terms were derived after consultation with the university librarian and discussion among authors. The search terms were applied to both abstracts and full texts during the searching process.

### Study Selection

To be included in this systematic review, all articles must (1) include children and adolescents within the age range of 5 to 18 years; (2) use smartphone technology as PA surveillance; (3) have specific PA behavioral outcomes including energy expenditure, steps count, and PA levels; (4) be written in English; and (5) be published between 2008 and 2023. The exclusion criteria were as follows: (1) participants out of age range; (2) not written in English; and (3) producing behavioral outcomes other than energy expenditure, steps, and PA levels (eg, sedentary behavior and sleep behavior).

### Data Extraction

All studies identified from the search were imported into the Endnote 20 referencing software. Endnote library was used for its invaluable reputation in managing records and keeping track of articles [34]. All records imported into Endnote included the following: title, authors, publication year, journal name, publisher, abstract, keywords, and the date on which it was searched. At this stage, all duplicates were removed using the software. All remaining records were then reviewed for applicability to scope based on the title and abstract and by referring to both the inclusion and exclusion criteria. Then, a full text of each remaining article was obtained and read to examine if it fully met the inclusion criteria and was therefore deemed fit for the data extraction process. In agreement with all authors, data extracted from the studies included study design, country, population, sample size, study duration, app name, app purpose, primary outcome, benefits of using the app, and limitations of using the app.

### Critical Appraisal of Evidence

In this systematic review, each study was assessed using the STROBE checklist for observational studies, which include cohort, case-control, and cross-sectional studies. This checklist was introduced to help produce a clear presentation of what was planned and performed in these types of studies. It consists of a checklist of 22 items, including the title, abstract, introduction, methods, results, and discussion section of the assessed study. In reference to Jain and Yuan’s review [35], the 22-items in the STROBE checklist were broken down into 47 distinct indicators for each marked study. A grading system was used based on the percentage of STROBE checklist criteria reported by each study: <55% was categorized as −, 55% to 65% was categorized as +, and ≥65% was categorized as ++. NINN reviewed the quality of all studies, with MEGA and JM reviewing 50% each. All authors met to discuss any discrepancies, and agreement was reached regarding the critical appraisal of each study.

## Results

### Study Selection

The selection process of all articles is shown in Figure S1 in Multimedia Appendix 2, documented in a Preferred Reporting Item for Systematic Reviews and Meta-Analyses study flow diagram. The database search yielded 584 unique and potentially relevant articles, including 15 articles from searching on Google Scholar, resulting in a total of 599 articles. After removing duplicates, 540 articles remained for title and abstract screening using the inclusion and exclusion criteria. At this stage, 482 articles were excluded for not meeting the inclusion criteria (268/482, 55.6%), not using smartphone technology for PA measurements (89/482, 18.5%), and not having relevant outcomes (125/482, 25.9%). This resulted in 10.7% (58/540) of articles that remained for full-text screening. Eight articles were included in this systematic review after the full-text screening (Multimedia Appendix 2). Initially, titles and abstracts were screened by the first author (NINN), with the other 2 authors (MEGA and JM) independently reviewing 50% each. Cohen κ was completed between NINN, MEGA, and JM, which showed an 89.3% agreement level (Cohen κ=0.89). The final selection of studies was performed after checking against the inclusion and exclusion criteria. This process involved thorough discussion among the authors until an agreement was reached.

### Study Characteristics

The included studies had a large sample size (6-492 participants), with a total sample of 881 children and adolescents, aged 9 to 18 years, as shown in Table 1. Of these, 5 studies involved healthy children and adolescents and the other 3 focused more on those who were overweight, obese, or had been diagnosed with type 1 diabetes mellitus. The studies were conducted in Germany (N=3) [36-38], Spain (N=1) [39], Australia (N=1) [40], the United States (N=1) [41], Sweden (N=1) [42], and Singapore (N=1) [43]. Most studies used a cross-sectional design (N=5) [40-43] or cohort design (N=3) [36-38]. The most common recruitment sites were primary care facilities [36-38] and schools [39-42], followed by 1 study involving volunteered children and adolescents [43]. As for the duration of the studies, there was a large range in the period of PA measurement, from 24 hours to 4 weeks.

**Table 1:** Characteristics of the studies (N=8).

Study		Country	Population	Sample size, n	Study duration	Quality of study^a^
**Cross-sectional**						
	Viciana et al [39], 2022	Spain	Children and adolescents (aged 12-18 years)	56	5 days	++
	Dahlgren et al [42], 2021	Sweden	Children and adolescents (age: mean 12.1, SD 1.5 years)	121	7 days	++
	Seah and Koh [43], 2020	Singapore	Adolescent girls (aged 15 years)	36	4 weeks	++
	Hartwig et al [40], 2019	Australia	Eighth-grade students (age: mean 13.5, SD 0.5 years)	492	35-50 minutes	++
	Dunton et al [41], 2014	United States	High school students (aged 15-18 years)	6	24 hours	+
**Cohort**						
	Schiel et al [36], 2012	Germany	Overweight and obese adolescents (age: mean 13.5, SD 2.8 years)	124	1-4 days	++
	Schiel et al [37], 2011	Germany	Children and adolescents with type 1 diabetes mellitus (age: mean 14.5, SD 2.2 years)	16	1-3 days	++
	Schiel et al [38], 2010	Germany	Overweight and obese adolescents (mean age 14.3 years)	30	1-4 days	++

^a^Percentage of Strengthening of the Reporting of Observational Studies in Epidemiology checklist criteria reported: +: 55%-65% and ++: >65%.

### Quality of Included Studies

In this systematic review, 5 studies were cross-sectional studies and 3 were cohort studies. All 8 studies were retrospective and critically appraised using the STROBE checklist. The percentage of indicators met by each study is shown in Figure S1 in Multimedia Appendix 3), and the overall quality is presented in Table 1. The STROBE checklist used in the critical appraisal process revealed that most of the included studies were of good quality. Items that were well reported across all studies included the following: title and abstract, background or rationale, quantitative variables, key results, limitations, and interpretation. However, there was less consistency across the studies in reporting aspects related to bias (3/8, 38%), study size (2/8, 25%), sensitivity analyses (2/8, 25%), reasons for nonparticipation at each stage (1/8, 13%), use of a flow diagram (1/8, 12%), number of participants with missing data (3/8, 38%), boundaries when continuous variables are categorized (1/8, 13%), other analyses done (eg, subgroups and sensitivity) (3/8, 38%), and generalizability (3/8, 38%).

### Smartphone Technology for PA Measurements

As stated previously, studies that were included used the app in smartphones or mobile phones as a surveillance tool for PA in children and adolescents. They either used a built-in and downloadable smartphone app from the Android Play Store and Apple App Store or used a study-specific developed smartphone app. In addition to using smartphone technology, several studies have added self-report questionnaires for PA recall [36,38,41,43] and accelerometers or pedometers [40,41,43], which in turn allowed comparisons between the methods.

Table 1 shows that the shortest period of PA measurement was 24 hours, which was conducted as a cross-sectional study [41]. Meanwhile, all 3 studies by Schiel et al [36-38] took 1-4 days for PA measurement in overweight and obese adolescents. The cross-sectional study by Viciana et al [39] was conducted for 1-5 days in adolescents aged 12-18 years. Another cross-sectional study conducted by Dahlgren et al [42] took approximately 7 days to measure PA in children and adolescents. Seah and Koh [43] measured the PA of high school adolescent girls for 4 weeks. The other 2 studies, instead of stating the overall time, focused more on the time spent for each measurement session. There were 3 groups involved in the study by Hartwig et al [40]: training sample, validation sample, and convergent validity. Each group had different periods of measurement: 68.7 (SD 22.2) minutes for the training sample, 67.6 (SD 21.6) minutes for the validation sample, and 47.0 (SD 0.7) minutes for the convergent validity group.

### Study-Specific Developed Apps

Five studies used a self-developed smartphone app designed specifically for the study [36-38,40,41], as shown in Table 2. Schiel et al [36-38] in all 3 studies used a self-developed technology comprising a mobile motion sensor (MoSeBo), which is a PA sensor, integrated into a mobile phone with a digital camera (DiaTrace), both developed by Fraunhofer Institut für Graphische Datenverarbeitung, Rostock, Germany. This app could analyze the type, intensity, and duration of PA. Each of the studies involved different participants but still focused on overweight and obese adolescents. Meanwhile, Dunton et al [41] used the Mobile Teen app, a self-developed smartphone technology that combines both objective and self-reported PA assessment through sensor-informed context-sensitive ecological momentary assessment and sensor-assisted end-of-day recall. Another study that used a specifically developed app was from Hartwig et al [40], in which the SmartLAB move+ app was used to measure feedback on the PA level achieved during physical education lessons.

**Table 2:** Summary of findings (study-specific developed apps).

Study	App name	App purpose	Primary outcome	Pros and cons
Hartwig et al [40], 2019	Custom-designed mobile app, wirelessly connected to a pedometer (SmartLAB move+)	Measure feedback on PA^a^ levels achieved during PE^b^ lessons	PA levels	Pros: feedback available immediately and increased PA during PE lesson Cons: translation of step counts to percent MVPA^c^. Available only for the particular study and not readily available for download from app stores
Dunton et al [41], 2014	Mobile Teen (installed on LG Nexus 4 smartphone)	The Mobile Teen app has 2 major components: sensor-informed CS-EMA^d^ and end-of-day sensor-assisted recall	EMA^e^ question sequence designed to measure major activity types, smartphone placement on the body, reasons for smartphone nonwear, and other psychological and contextual factors related to behavior	Pros: records phone location and useful in providing bouts of a specific type of behavior Cons: compatible with Android phones only and EMA prompts >1 in an hour. Available only for the particular study and can only be used in Android phones
Schiel et al [36], 2012	MoSeBo and DiaTrace system (motion sensor board with a digital camera)	Analyze type, intensity, and duration of PA	Type, intensity, and duration of PA	Pros: accurate measurement of PA (time, intensity, and duration) compared with a self-report questionnaire Cons: available only for the particular study and not readily available for download from app stores
Schiel et al [37], 2011	MoSeBo and Diatrace system (motion sensor board with a digital camera)	Analyze type, intensity, and duration of PA	Type, intensity, and duration of PA	Pros: visualization of PA and real-time display Cons: available only for the particular study and not readily available for download from app stores
Schiel et al [38], 2010	MoSeBo and Diatrace system (motion sensor board with a digital camera)	Analyze type, intensity, and duration of PA	Type, intensity, and duration of PA	Pros: more accurate measurement compared with questionnaire and improves both intrinsic and extrinsic motivation Cons: unable to measure water-based activities. Available only for the particular study and not readily available for download from app stores

^a^PA: physical activity.

^b^PE: physical education.

^c^MVPA: moderate to vigorous physical activity.

^d^CS-EMA: context-sensitive ecological momentary assessment.

^e^EMA: ecological momentary assessment.

### Readily Downloadable Apps

Three studies used readily downloadable apps from Apple App Store and Android Play Store. In the study by Viciana et al [39], 4 apps were used including Pedometer and Pacer Step Counter, which can be downloaded from the app store, whereas Google Fit and Apple Health were 2 built-in apps in the Android and iOs systems. Meanwhile, the SCRIIN app was used in the study by Dahlgren et al [42] to measure PA in children and adolescents, alongside the SCRIIN activity tracker. In the study by Seah and Koh [43], several apps were used, including MapMyFitness, Health (Apple), Samsung Health, Pacer Step Counter, Pedometer, and Weight Loss Coach. All these apps were used to track PA behaviors and step counts.

### The Primary Outcome of Each App

All smartphone and mobile phone apps from the studies included in this review had the same primary outcome, that is, measuring PA. As shown in Tables 2 and 3, although all apps are used to measure PA, each app has a different focus and function. The apps that were built purposely for the study have specific functions to answer questions specific to the study. In the study by Hartwig et al [40], the primary outcome of the SmartLAB move+ app was the PA level. The MoSeBo and Diatrace system in the studies by Schiel et al [36-38] also produced results on PA intensity, in addition to PA type and duration. In contrast to other studies, the study by Dunton et al [41] used a sensor-informed context-sensitive ecological momentary assessment–measured type of PA only, but this app also produced results regarding smartphone placement on the body, reasons for smartphone nonwear, and other psychological and contextual factors related to behavior.

**Table 3:** Summary of findings (readily downloadable apps).

Study	App name	App purpose	Primary outcome	Pros and cons
Dahlgren et al [42], 2021	SCRIIN activity tracker	Measure PA^a^	Active minutes and steps count	Pros: SCRIIN activity tracker can be purchased; SCRIIN app can be downloaded via Apple App Store and Google Play Store Cons: missing and incompleteness of data from SCRIIN app owing to children’s and adolescents’ need to have access to a smartphone
Viciana et al [39], 2022	Pedometer, Pacer Step Counter, Google Fit, Apple Health	Measure PA aspects and step count	Step counts and PA in free-living conditions	Pros: all apps are built-in and can be downloaded from App Store. Some of the apps have been used in previous studies Cons: apps are all not empirically validated
Seah and Koh [43], 2020	MapMyFitness (for PA), Apple Health, Samsung Health, Pacer Step Counter, Pedometer, Weight Loss Coach (for step count)	Track PA aspects and step count	Duration, distance, pace, speed, elevation, calories burned, and route traveled	Pros: quick feedback and easy for self-monitoring Cons: apps are all not empirically validated

^a^PA: physical activity.

Most existing and downloadable apps have specific functions that produce specific PA outputs such as energy expenditure, step counts, and PA level. The SCRIIN app used in the study by Dahlgren et al [42] can produce results on active minutes and step count. In the study by Seah and Koh [43], several apps were used to measure PA (MapMyFitness) and step count (Health, Samsung Health, Pacer Step Counter, Pedometer, and Weight Loss Coach) differently, but the outcomes from all the apps were PA duration, distance, pace, speed, elevation, calories burned, and route traveled. Apps used in the study by Seah and Koh [43] were similar to those used in the study by Viciana et al [39], in which Pedometer and Pacer Step Counter were used to measure step counts and Google Fit and Apple Health were used to track both energy expenditure and step counts.

### Pros and Cons of Each App

When considering the usefulness of different methods to measure PA in children and adolescents, it is important to highlight the common pros and cons reported across the included studies. The first advantage of smartphone apps is the additional features available when measuring PA. For example, the SmartLAB move+ app [40] and all apps used in the studies by Seah and Koh [43] and Viciana et al [39] provided immediate feedback to participants during PA measurement. This allowed research participants to have personal health monitoring, in which they could easily access their PA data at any time. In comparison with research-grade accelerometers, raw data produced by accelerometers will have to be translated before being transformed into PA summaries (eg, calories and step counts) [44]. Furthermore, the Mobile Teen app offers a feature that records mobile phone location, which is useful for determining where PA took place [41].

A second advantage is the accessibility of the apps used in studies, where using the apps is a low-cost option and the apps are easily downloadable from Apple App Store and Google Play Store. For example, the SCRIIN app that was used in the study by Dahlgren et al [42] and all apps used in the study by Seah and Koh [43] are readily available and can be downloaded via Apple App Store and Android Play Store. In addition, these 2 studies revealed that users are more interested in fun, easy-to-use, and functional apps that offer visual appeal, as highlighted in a previous study by Schoeppe et al [45]. Considering that the target participants are children and adolescents, fun and user-friendly apps will assist in the PA monitoring of this specific age group [46].

The third advantage is the usability of all apps, which means that the apps used are easily downloadable into users’ smartphones, easy to use, and enable young users such as children and adolescents to self-monitor their PA measurements. However, it is worth mentioning that the advantages of all apps discussed are heavily dependent on the studies included in this review. In terms of usability of the apps among research participants, the study by Seah and Koh [43] revealed that participants preferred to have more control over the PA data while using the apps, including setting their own goals and social connectivity. A similar response was reported in the study by Viciana et al [39], in which the usability of the apps allowed participants to monitor and control the apps on their own and enabled them to view previous activities. For researchers, data generated through apps that can be easily obtained instantly was noted as a strength [47].

In all the studies by Schiel et al [36-38], the MoSeBo and Diatrace system demonstrated a better visualization of PA and real-time display, and the system has been shown to provide more accurate measurements (time, intensity, and duration) than the self-report questionnaire. Meanwhile, the Mobile Teen app is useful in providing bouts of PA performed by the participants [41], a feature similar to accelerometers, providing greater detail regarding the times spent in different PA intensities. Furthermore, the SCRIIN app used in the study by Dahlgren et al [42] showed a high correlation (*r*=0.72; *P*<.001) between the SCRIIN activity tracker and app with the ActiGraph accelerometer in the validation they conducted. This showed that the app is a valid measure for PA monitoring in children and adolescents, when compared with accelerometry.

Nevertheless, all apps also have disadvantages, especially in the accessibility of apps, validity, and practicality in measuring PA. Regarding the apps’ accessibility, some apps were specifically developed for the studies and were not downloadable via Apple App Store and Google Play Store [36-38,40,41]. In addition, some apps have not been properly validated for measuring PA in children and adolescents. In the studies by Seah and Koh [43] and Viciana et al [39], although all the apps used were free and convenient for measuring PA in participants, they were not empirically validated. It was also mentioned in Seah and Koh’s study [43] that caution should be taken when reading and interpreting the results.

In addition, the SmartLAB move+ app used in the study by Hartwig et al [40] requires researchers to translate step counts to percent MVPA using an equation that is unlikely to be generalizable to populations other than those tested in this study. This factor affects the usability of this app to researchers, where researchers will have to do the *extra work* rather than obtaining the readily available PA data directly from the app. Meanwhile, the MoSeBo and Diatrace system used in all the studies by Schiel et al [36-38] is unable to measure water-based activities. In terms of the practicality of the apps, a con of the Mobile Teen app is its compatibility with Android phones only, which will limit its use [41].

Finally, it was mentioned that real-time feedback is a strength of these apps but could also create a potential Hawthorne effect (leading to unexpected changes in behavior) because participants know their behavior is being measured [48]. In this case, being able to see PA levels may cause a person to be more active because they know that PA is being assessed [49]. This leads to a recommendation to assess the validity of these apps against known criterion measures of PA, for instance, smartphone apps versus research-grade accelerometry.

## Discussion

### Smartphone Technology in PA Surveillance

This systematic review identified and examined 8 articles on the use of smartphone technology in PA surveillance of children and adolescents globally. This review specifically focuses on smartphone or mobile phone apps that can be used for PA surveillance among children and adolescents. The low number of studies included in this review indicates limited research on using smartphone apps for PA surveillance in children and adolescents. Nonetheless, there are existing studies on adults who have used smartphone apps for PA surveillance [50-54]. As smartphones are considered a *must-have* item nowadays and are often carried throughout the day, this allows them to be a method to measure and monitor PA in real time [55]. Currently, new apps are being developed that allow users to track and monitor their PA using their smartphones, increasing the possibility of obtaining richer objective PA profiles, which will complement the traditional or subjective methods of PA measurement [56]. Some included studies used specifically designed apps for measuring PA among children and adolescents, which may be because of the limited number of commercially available smartphone apps for PA surveillance at the time.

As mentioned earlier, smartphone and mobile phone apps were first introduced between 2008 and 2012. However, as subjective assessments are more widely used [49,57,58], the number of studies using apps for PA surveillance in children and adolescents remains limited [41,56,59]. As seen from the results of this review, most of the current studies (after 2019) used readily downloadable smartphone apps to measure PA in children and adolescents. The advancement of smartphone technology over the years has influenced the development of PA monitoring apps in smartphones, thus allowing researchers to use these apps in their validation studies of PA measurement in children and adolescents [56,60,61].

Another important finding is that all studies in this review were conducted in high-income countries, including Australia, the United States, Greece, Sweden, and Singapore [62]. This highlights the progressive development of the mobile phone and smartphone industry in high-income countries, which allows the use of smartphone technology for PA surveillance among children and adolescents [63]. The recent evolution of smartphones has resulted in the emergence of numerous apps with novel ways to promote healthier lifestyles, including measuring and monitoring PA [52]. However, this point also crucially shows the lack of PA research using smartphone technology in low- and middle-income countries (LMICs).

In 2017, a group of researchers from Stanford University conducted a study using smartphone data to track PA among adults globally, specifically using the free Azumio Argus smartphone app [64]. This study revealed that from the 46 countries involved with at least 1000 users, 90% of the users were from 32 high-income countries and only 10% of the users were from 14 middle-income countries [64]. However, there were none from low-income countries. This showed that people in high-income countries are more exposed to smartphone apps that can be used to track their PA. Another important finding from this study is that countries with high PA inequality (ie, the gap between highly active people and less active people) also have high obesity rates. The PA levels used to determine PA inequality were calculated from the PA data collected using the smartphone app [64].

In their systematic review, Bort-Roig et al [56] also revealed that all studies that used smartphone technology to track PA in adults were conducted in highly economically advantaged countries, where most of the studies were conducted in the United States, Germany, and Finland. Measurements of PA in this review included smartphone apps with built-in accelerometers and pedometers, and some studies used wearable activity trackers to pair with the apps. This finding strengthens the point that PA research using smartphone technology to track PA in children and adolescents is widely conducted in high-income countries; however, it is still lacking in LMICs, particularly.

### Smartphone Technology Versus Other PA Objective Measures

As stated in a review by Tudor-Locke and Myers [65], there are various methods of PA measurement that are often categorized into subjective and objective measures. For children and adolescents, objective measures of PA include direct observation, direct and indirect calorimetry, doubly labeled water, heart rate monitoring, and the use of motion sensors such as pedometers and accelerometers. Pedometers and research-grade accelerometers have been widely used to measure step counts and PA in children and adolescents [16,66-68]. Some researchers have used pedometers for PA surveillance in children and adolescents, as they are low cost, more feasible, and have been shown to be reliable and valid in school children and adolescents [69-71].

However, pedometers may underestimate vigorous-intensity activities, which in contrast can be more accurately measured by accelerometers [72,73]. Objectivity and low subject reactivity are pros of accelerometers, characteristics that overcome some of the challenges with subjective measures [73]. Moreover, accelerometers have been shown to provide valid measures of PA in school children [73]. Nevertheless, it is important to note that research-grade accelerometers are expensive and may not be affordable for everyone.

In this particular review, smartphone apps used to measure PA and step counts have the same principle of objective measurement as pedometers and research-grade accelerometers; however, they are downloadable onto smartphones. This important feature allows users to have a personal health monitoring device, which provides better compliance data and continuous evaluation of free-living activities [55]. It is also important to note that smartphones have multiple built-in sensors and capabilities that include large memory storage, fast processors, and microelectromechanical systems, facilitating a better opportunity for PA measurement [55].

In addition, for research that uses smartphone apps and technology, potential participants will already own this *research device* and are more likely to carry it wherever they go and keep it charged, which will be a different situation to devices handed out in research studies [55]. The advantages of smartphone apps and technology include their availability (free or low cost, provided the research participant owns a smartphone), accessibility (downloadable), quick feedback, low cost, and ease of monitoring, which influenced the 3 studies included in this review to use them for PA measurement of children and adolescents [40-43].

### Potential Research Gap

This systematic review has highlighted 2 important points for future studies. First, it was demonstrated throughout this review that smartphone apps and technology are a potential alternative for the objective measurement of PA among children and adolescents. As mentioned in the *Discussion* section, objective measures would benefit younger people and allow a more feasible assessment that can include numerous dimensions and domains of PA. Second, this review identified conflicting evidence on the validity and reliability of smartphone apps in measuring PA in children and adolescents. This shows a lack of evidence owing to the dearth of individual primary studies that assess the psychometric properties of smartphone apps.

Apart from having more validation studies involving smartphone apps and technology in PA surveillance in this age group, it is equally important to include subjective measures. This is because the use of combined measures may offer a better comparison between the 2 and provide a better understanding of the characteristics of PA in children. On the basis of a review by Troiano et al [74], it was revealed that accelerometer-based devices (where smartphone apps may fall in this category) are more accurate in measuring self-reported PA variables such as frequency and duration in comparison with using a series of questions in questionnaires. However, it is unfitting to conclude that PA measures using accelerometer-based devices are better than using questionnaires. Each approach has complementary strengths, but it is important to note that behavioral reports and device-based measures are not interchangeable [74].

Apart from these points, no studies have assessed the usability of smartphone apps for PA surveillance among children and adolescents in LMICs. All studies included in this review were conducted in high-income countries, raising the question of whether socioeconomic status influences the use of smartphone apps and technology in PA measurement. Understanding the use of smartphones among children and adolescents in LMICs could further highlight the potential of using smartphone apps as PA surveillance tools in this setting.

Despite its numerous advantages, it is undeniable that smartphone apps and technology also has some limitations. One of the notable limitations of smartphone apps and technology is the proprietary algorithms used in the apps, commonly known as nonfree or closed-source algorithms, which restrict users’ freedom to obtain data collected through the apps [75]. This type of algorithm may require researchers to have proper permission or licensing from app developers and manufacturers to obtain collected data [75,76].

### Strengths and Limitations

A systematic approach was used to identify articles on PA surveillance using smartphone technology for children and adolescents. It was conducted using several databases that were relevant to the research topic, which further strengthened this review. Another advantage of this review was the use of independent screening conducted by 3 researchers. However, the searching process only included articles written in English, which may limit the potential of exploring established studies on PA surveillance in children and adolescents that were not published in English. This factor may also have affected the lack of studies from LMICs included in this review owing to the use of languages other than English. In addition, the focused research questions resulted in a small number of articles being included in this review. Another important limitation to note is the fact that smartphone apps are continuously evolving and being created, meaning findings from this review, although useful, might not apply to the most recent apps.

### Conclusions

The overall results from this review demonstrate conflicting and insufficient evidence regarding the validity and reliability of smartphone technology PA surveillance in children and adolescents. This review also suggests that additional research is needed to further assess the usability and usefulness of smartphone technology for PA surveillance in children and adolescents. This is especially important for LMICs.

## Appendix

Multimedia Appendix 1 Detailed search strategy applied to all databases.

Multimedia Appendix 2 Summary of the selection of the articles presented in the PRISMA (Preferred Reporting Items for Systematic Reviews and Meta-Analyses) flow diagram.

Multimedia Appendix 3 Critical appraisal of the included studies.

## Abbreviations

LMIC: low- and middle-income country
mHealth: mobile health
MVPA: moderate to vigorous physical activity
PA: physical activity
PRISMA: Preferred Reporting Items for Systematic Reviews and Meta-Analyses
STROBE: Strengthening of the Reporting of Observational Studies in Epidemiology
