# Supplementary material for: Physical Activity Surveillance in Children and Adolescents Using Smartphone Technology: Systematic Review
Source: JMIR Pediatr Parent. 2023 Mar 29;6:e42461. doi: 10.2196/42461 (PMC10131756; doi:10.2196/42461)
Supplement: Multimedia Appendix 1 [file pediatrics_v6i1e42461_app1.docx]

## Multimedia Appendix 1

## Detailed search strategy applied to all databases

smartphone* OR “smartphone app*” OR “mobile phone” OR “mobile app*” OR “smartphone technolog*” OR “mobile technolog*”

AND

“physical activity” OR “physical activity level” OR “step count*” OR “energy expenditure*” OR exercise

AND

child* OR adoles* AND measurement* OR assessment* OR surveillance
