# Supplementary material for: Physical Activity Surveillance in Children and Adolescents Using Smartphone Technology: Systematic Review
Source: JMIR Pediatr Parent. 2023 Mar 29;6:e42461. doi: 10.2196/42461 (PMC10131756; doi:10.2196/42461)
Supplement: Multimedia Appendix 2 [file pediatrics_v6i1e42461_app2.docx]

## Multimedia Appendix 2


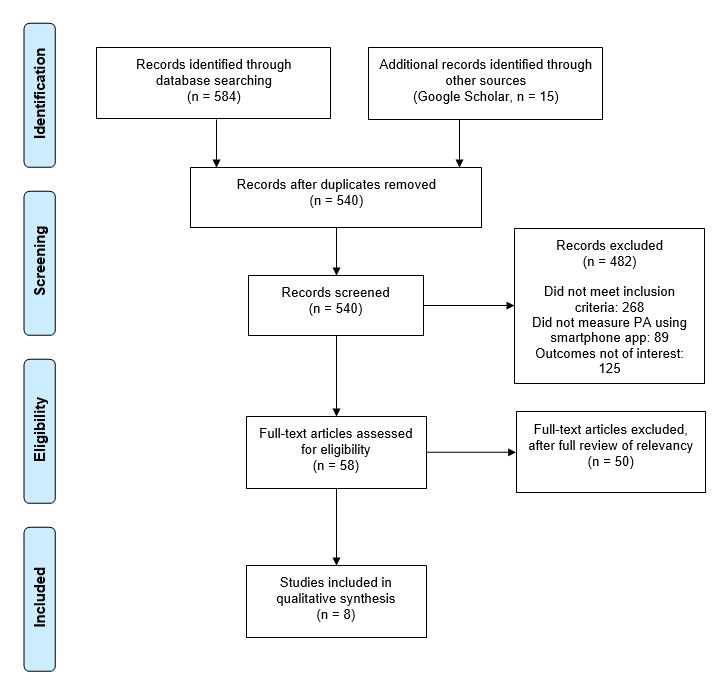


Figure 1 Summary of the selection of the articles presented in the PRISMA Flow Diagram
