# Supplementary figures and images for: Physical Activity Surveillance in Children and Adolescents Using Smartphone Technology: Systematic Review
Source: JMIR Pediatr Parent. 2023 Mar 29;6:e42461. doi: 10.2196/42461 (PMC10131756; doi:10.2196/42461)

## Multimedia Appendix 3


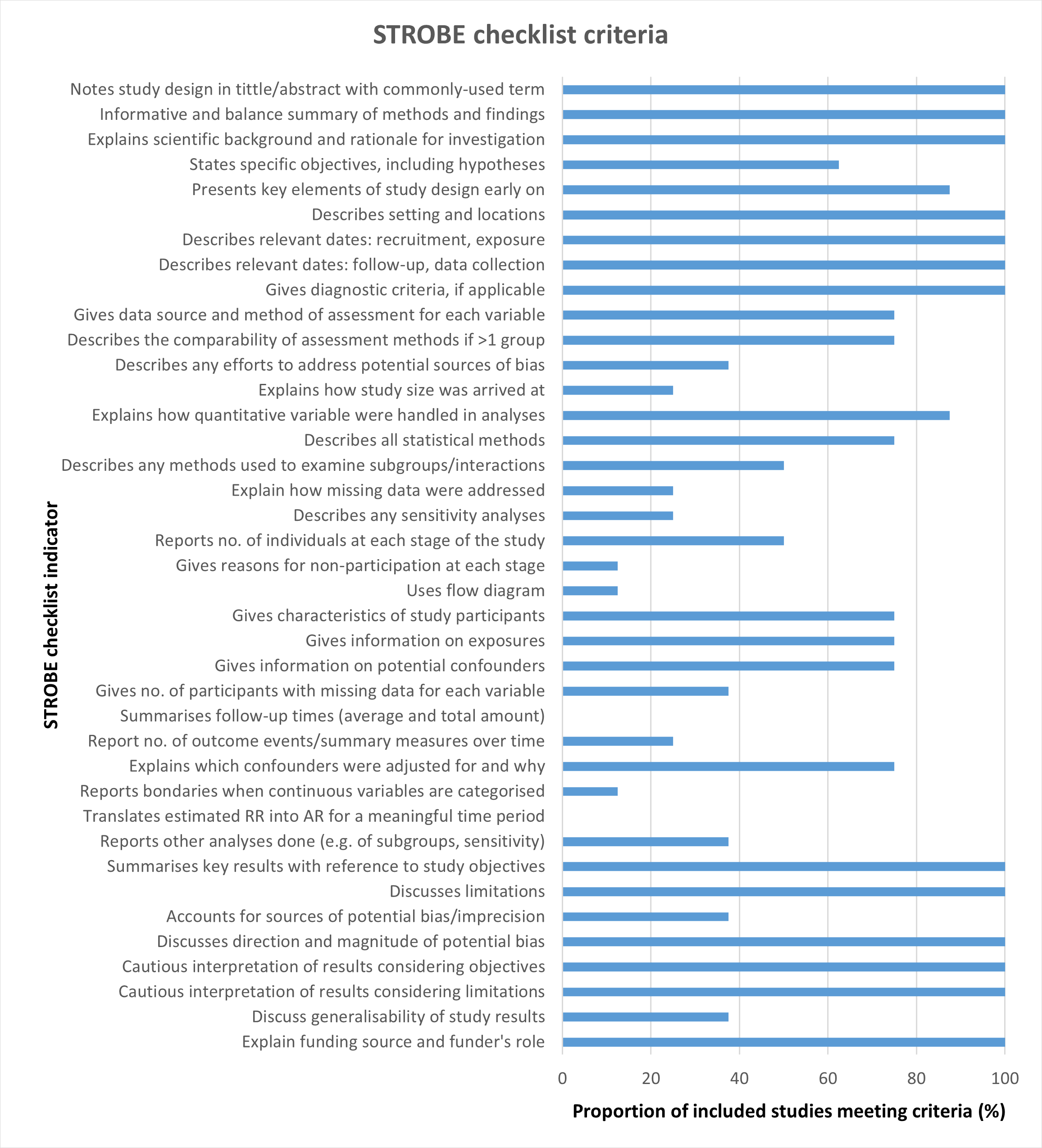


Figure 2 Critical appraisal of included studies

Supplement: Multimedia Appendix 3 [file pediatrics_v6i1e42461_app3.docx]
